# Death and Graft Loss in Simultaneous Pancreas-Kidney Recipients by Donor-Recipient Cytomegalovirus Serostatus in the United States

**DOI:** 10.3389/ti.2025.15653

**Published:** 2025-11-26

**Authors:** Jose Arriola-Montenegro, Byron H. Smith, Naim S. Issa, Aleksandra Kukla, Yogish C. Kudva, Paul J. Deziel, Mikel Prieto, Raymund R. Razonable, Samy M. Riad

**Affiliations:** 1 Department of Medicine, Division of Nephrology and Hypertension, Rochester, MN, United States; 2 Mayo Clinic School of Graduate Medical Education, Mayo Clinic College of Medicine and Science, Rochester, MN, United States; 3 Division of Clinical Trials and Biostatistics, Rochester, MN, United States; 4 Department of Medicine, Mayo Clinic William J. von Liebig Center for Transplantation and Clinical Regeneration, Rochester, MN, United States; 5 Department of Surgery, Division of Infectious Diseases, Rochester, MN, United States; 6 Department of Medicine, Mayo Clinic, Rochester, MN, United States; 7 Division of Endocrinology, Diabetes, Metabolism, and Nutrition, Department of Medicine, Rochester, MN, United States

**Keywords:** simultaneous kidney pancreas transplantation, CMV serostatus, D+/R-, donor cytomegalovirus positive, recipient negative

## Abstract

Cytomegalovirus (CMV) serologic discordance is a known risk factor for adverse outcomes after solid-organ transplantation. This study evaluated outcomes of simultaneous pancreas–kidney (SPK) recipients based on donor and recipient CMV serostatus. Using the Scientific Registry of Transplant Recipients, we identified adult SPK recipients between 2014 and 2024 and categorized them as donor/recipient negative (D−/R−), recipient positive (R+), or donor positive/recipient negative (D+/R−). Patients with missing data, nonstandard immunosuppression, or positive crossmatch were excluded. Among 4,744 recipients (831 D−/R−, 2,671 R+, 1,242 D+/R−), the D+/R− group had the highest 1-year rates of graft rejection (16.6%, p = 0.02) and hospitalization (67.2%, p = 0.005), whereas the D−/R− group had the lowest (11.8% and 60.0%, respectively). In multivariable models, D+/R− recipients had higher risks of death (HR 1.28; 95% CI, 1 .01–1.62; p = 0.045), pancreas graft-loss (HR 1.25; 95% CI, 1.06–1.48; p = 0.009), and death-censored kidney graft-loss (HR 1.31; 95% CI, 1.01–1.69; p = 0.04) compared with R+. Conversely, D−/R− recipients had a lower risk of kidney graft-loss (HR 0.66; 95% CI, 0.46–0.96; p = 0.03). CMV D+/R− serostatus is independently associated with increased mortality and graft-loss after SPK transplantation. Matching CMV-seronegative donors with seronegative recipients may improve outcomes, warranting further study of the feasibility and broader impact of CMV serostatus–based-matching.

## Graphical Abstract

**Figure:**
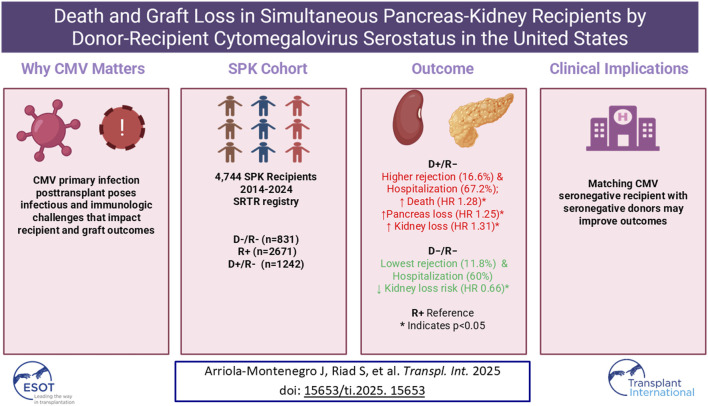

## Introduction

Simultaneous pancreas-kidney (SPK) transplant offers persons with diabetes-related uremia freedom from both dialysis and insulin dependence [1], with the additional potential to reverse microvascular disease [2] over time. Moreover, compared with kidney-alone transplant [3–7], SPK transplant offers substantial survival and quality-of-life benefits and is the most cost-effective option when considering patient and graft survival probabilities [8]. Despite these advantages, SPK transplant presents several challenges related to immunologic and infectious complications [9, 10].

One challenge for SPK transplant recipients is the pancreas graft’s high immunogenicity [11–14], which requires augmented immunosuppression to mitigate the risk of rejection [15, 16] and makes SPK recipients more susceptible than kidney-only recipients to complications such as cytomegalovirus (CMV) infection, especially in cases involving a donor who is CMV positive and a recipient who is CMV negative (D+/R–) [17–22]. CMV infection, a common and serious complication in solid-organ transplant recipients, increases the incidence of hospitalization, graft loss, and death among kidney transplant recipients, particularly among those who underwent SPK transplant [23, 24]. CMV infection is most prevalent in recipients who do not receive antiviral prophylaxis or who are exposed to high doses of immunosuppressive therapy [17, 24]. The risk of primary CMV infection is particularly elevated for D+/R− cases [19, 25, 26], which occur frequently with SPK transplant [27, 28].

Prophylactic administration of valganciclovir is the standard of care for all SPK patients who are at risk for CMV primary infection or reactivation (recipient positive, R+, or donor positive and recipient negative, D+/R–) and has proved beneficial in reducing rates of CMV infection after SPK transplant [29]. This preventive intervention has been shown to improve both short- and long-term allograft outcomes in SPK recipients by reducing the incidence of CMV-related complications [17, 29, 30]. However, valganciclovir does not entirely prevent CMV infection; in one study, up to 38% of kidney transplant recipients had delayed-onset primary CMV infection after completing 6 months of valganciclovir prophylaxis [31].

Given these considerations, the current analysis aimed to assess the long-term outcomes of SPK transplant recipients on the basis of donor-recipient CMV risk profiles and shed light on the potential effect of CMV serostatus discordance on recipient and graft survival. We analyzed the outcomes of a contemporary cohort of SPK recipients, who were of average immunologic risk, by donor-recipient CMV serostatus.

## Materials and Methods

### Data Source

This study used data from the Scientific Registry of Transplant Recipients (SRTR). The SRTR data system includes data on all donor, wait-listed candidates, and transplant recipients in the US submitted by the members of the Organ Procurement and Transplantation Network (OPTN). The Health Resources and Services Administration (HRSA), U.S. Department of Health and Human Services provides oversight to the activities of the OPTN and SRTR contractors. The study was deemed exempt by the Mayo Clinic Institutional Review Board (INC8014532).

### Study Population

In the SRTR standard analysis file we identified all patients who received an SPK transplant between 1 January 2014, and 31 March 2024. During the study period, valganciclovir prophylaxis was routinely administered to SPK recipients for 3 months for R+ cases or up to 6 months for D+/R− cases. CMV-naïve recipients with a CMV-negative donor (D−/R−) did not receive valganciclovir prophylaxis [17, 23]. We excluded recipients on the basis of induction regimen (missing; mixed; or other than rabbit anti-thymocyte globulin, alemtuzumab, and interleukin-2 receptor agonist), maintenance regimen (missing, or other than tacrolimus and mycophenolate mofetil with or without corticosteroids), crossmatch data (missing, positive, or weakly positive), and CMV serostatus (missing). We categorized recipients into three risk groups on the basis of recipient and donor CMV serostatus: low-risk, D−/R−; intermediate-risk, R+; and high-risk, D+/R−, consistent with the risk stratification endorsed by major transplant and infectious diseases societies.

### Outcomes of Interest

The primary outcomes of interest were recipient and overall allograft survival by donor-recipient CMV serostatus risk category. Death-censored allograft survival was also evaluated. Short-term outcomes included 1-year rates of hospitalization, rejection of kidney alone, rejection of pancreas alone, and rejection of either organ.

### Statistical Analyses

Continuous variables were summarized as means and SDs and compared by using analysis of variance or pooled *t* tests. Categorical variables were summarized as counts and percentages and compared with the χ^2^ test.

Time-to-event data were summarized with Kaplan-Meier estimates of incidence through 7.5 years post transplant. The log-rank test was used to compare groups. Cox proportional hazards models (referred to as *multivariable models*) were used to evaluate the effect of CMV serostatus risk category on outcomes of interest, with adjustment for the following possible confounding variables: age, sex, ethnicity, diabetes type, preemptive transplant, dialysis duration, induction type, corticosteroid maintenance, HLA antigen mismatch, calculated panel reactive antibody, local vs. imported organs, pancreas donor risk index, transplant year, and donor-recipient Epstein-Barr virus status. The center was entered as a random effect in the multivariable models. Linearity in all tests was evaluated by using splines for continuous variables. We used Schoenfeld residuals plots to test the assumption of proportionality.

All analyses were performed with R version 4.2.2 (R Foundation for Statistical Computing). Statistical significance was defined as *P* < 0.05.

## Results

### Baseline Characteristics

**FIGURE 1:**
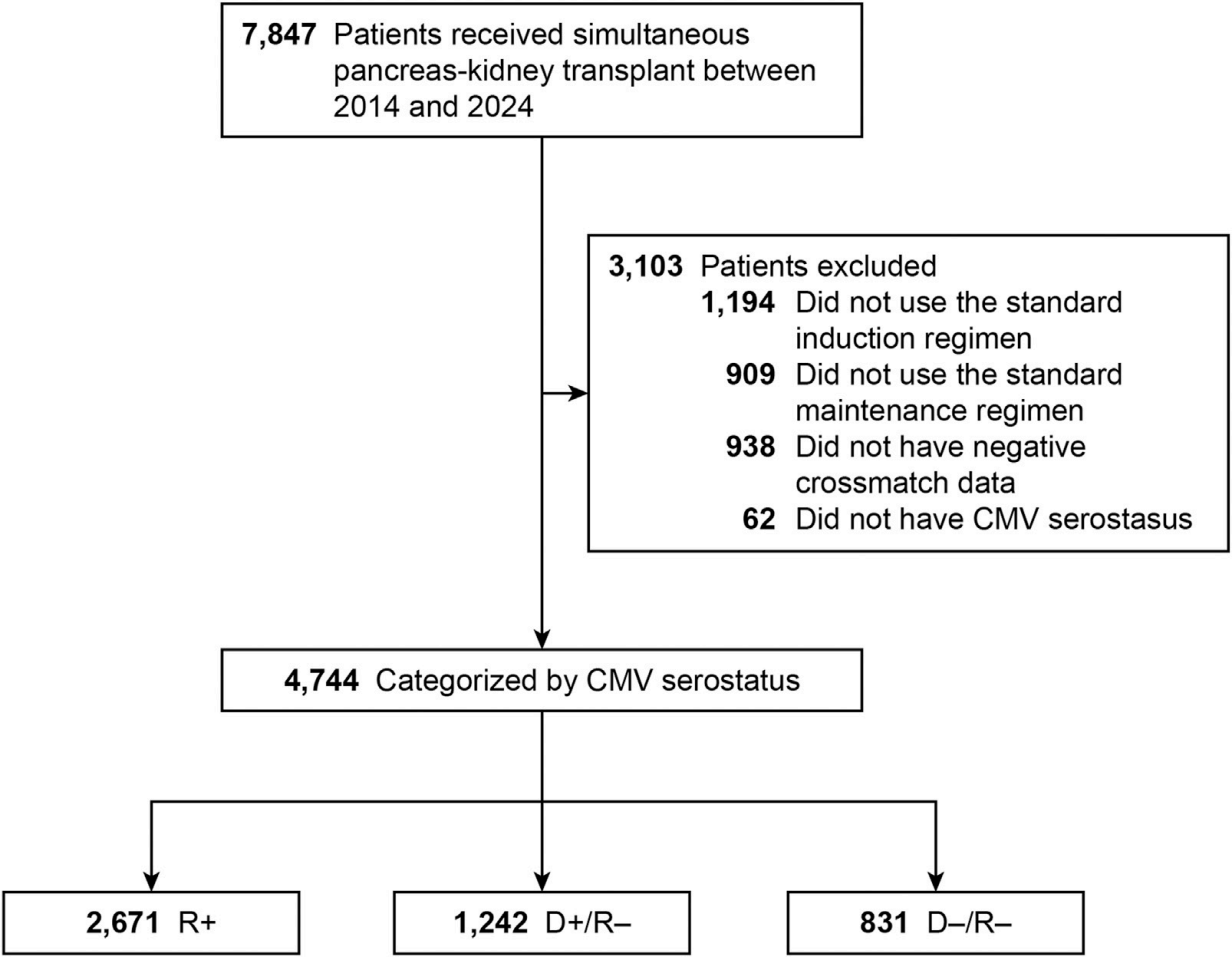
Flowchart of Study Design. Flowchart of study design showing inclusion and exclusion criteria for simultaneous pancreas–kidney (SPK) transplant recipients categorized by donor–recipient cytomegalovirus (CMV) serostatus. CMV indicates cytomegalovirus; R+, CMV-positive transplant recipients; D+/R–, CMV-positive donors and CMV-negative recipients; D–/R–, CMV-negative donors and recipients.

We identified 7,847 patients who underwent SPK transplant during the study period. The final analysis cohort consisted of 4,744 SPK recipients with complete data: 831 low-risk (D−/R−), 2,671 intermediate-risk (R+), and 1,242 high-risk (D+/R−) (Figure 1).

Table 1 details the demographic characteristics of recipients and donors by CMV serostatus. Recipients had a mean (SD) age of 42.3 (9.2) years and body mass index (calculated as weight in kilograms divided by height in meters squared) of 25.7 (4.2); these features were similarly distributed across risk groups. Overall, 61.6% of recipients were men, but the R+ group had a significantly lower share of men than the other groups (*P* < 0.001). Black and Hispanic recipients were significantly more represented in the R+ group (*P* < 0.001).

**TABLE 1:** Baseline characteristics of recipients, donors, and transplants^a^.

Characteristic	D−/R− (N = 831)	R+ (N = 2,671)	D+/R− (N = 1,242)	*P* value
Recipients				
Age, y	42.0 (9.1)	42.3 (9.1)	42.4 (9.5)	0.58
Sex				<0.001
Female	259 (31.2)	1,166 (43.7)	398 (32.0)	
Male	572 (68.8)	1,505 (56.3)	844 (68.0)	
Ethnicity				<0.001
White	530 (63.8)	1,104 (41.3)	797 (64.2)	
Black	202 (24.3)	890 (33.3)	290 (23.3)	
Hispanic	72 (8.7)	505 (18.9)	117 (9.4)	
Other	27 (3.2)	172 (6.4)	38 (3.1)	
BMI	25.60 (4.40)	25.79 (4.18)	25.65 (4.10)	0.43
Dialysis duration, y	1.8 (1.9)	2.1 (2.0)	1.8 (1.8)	<0.001
EBV status	n = 760	n = 2,349	n = 1,124	<0.001
R+	655 (86.2)	2,164 (92.1)	1,007 (89.6)	
R−/D+	76 (10.0)	163 (6.9)	107 (9.5)	
R−/D−	29 (3.8)	22 (0.9)	10 (0.9)	
Diabetes type	n = 828	n = 2,640	n = 1,234	<0.001
Type 1	709 (85.6)	2,023 (76.6)	1,022 (82.8)	
Type 2	119 (14.4)	617 (23.4)	212 (17.2)	
Preemptive transplant	151 (18.2)	402 (15.1)	240 (19.4)	0.002
Peripheral vascular disease	125 (15.1)	319 (11.9)	172 (13.9)	0.07
Donors				
Age, y	23.4 (8.1)	24.3 (7.9)	24.6 (7.7)	0.002
Ethnicity				<0.001
White	608 (73.2)	1,579 (59.1)	712 (57.3)	
Black	135 (16.2)	593 (22.2)	267 (21.5)	
Hispanic	62 (7.5)	401 (15.0)	219 (17.6)	
Other	26 (3.1)	98 (3.7)	44 (3.5)	
Sex				0.76
Female	242 (29.1)	799 (29.9)	358 (28.8)	
Male	589 (70.9)	1,872 (70.1)	884 (71.2)	
PDRI	0.98 (0.24)	0.99 (0.25)	0.98 (0.24)	0.97
Local organs	579 (69.7)	1,801 (67.4)	829 (66.7)	0.35
Non–heart-beating donor	32 (3.9)	73 (2.7)	19 (1.5)	0.004
Transplants				
Calculated PRA, %	11.3 (22.8) (n = 792)	15.6 (26.9) (n = 2,487)	11.9 (23.7) (n = 1,170)	<0.001
No. of HLA antigen mismatches	4.57 (1.12)	4.66 (1.09)	4.58 (1.10)	0.05
Induction type				0.01
r-ATG	636 (76.5)	2,083 (78.0)	970 (78.1)	
Alemtuzumab	145 (17.4)	497 (18.6)	211 (17.0)	
IL-2RA	50 (6.0)	91 (3.4)	61 (4.9)	
Corticosteroid maintenance	583 (70.2)	1,848 (69.2)	902 (72.6)	0.09
Length of hospitalization, d	9.9 (10.1) (n = 831)	9.9 (11.7) (n = 2,666)	9.8 (8.4) (n = 1,242)	0.95

Abbreviations: BMI, body mass index; D–/R–, CMV-negative donors and recipients; D+/R–, CMV-positive donors and CMV-negative recipients; EBV, Epstein-Barr virus; IL-2RA, interleukin-2 receptor agonist; PDRI, pancreas donor risk index; PRA, panel reactive antibody; r-ATG, rabbit anti-thymocyte globulin; R+, CMV-positive recipients.

^a^ Values are mean (SD) or No. of patients (%).

Recipients in the R+ group were significantly less likely than those in the D–/R–and D+/R–groups to receive a preemptive transplant (15.1% vs. 18.2% vs. 19.4%, respectively) (*P* = 0.002) and were on dialysis for significantly longer than recipients in the other groups (mean, 2.1 vs. 1.8 vs. 1.8 years; *P* < 0.001). The pancreas donor risk index and proportion of locally procured organs did not differ between groups. The proportion of organs procured after circulatory death was significantly higher in the D–/R–group than in the R+ and D+/R–groups (3.9% vs. 2.7% vs. 1.5%, respectively; *P* = 0.004). The groups also differed significantly in terms of diabetes type, preemptive transplant, calculated panel reactive antibody, induction type, Epstein-Barr virus status, and donor age and ethnicity (Table 1).

### Univariable Outcomes

One-year outcomes post transplant are shown in Table 2. Kidney rejection rates and pancreas rejection rates did not differ significantly between risk groups, but the combined kidney or pancreas rejection rate did. The combined rejection rate was significantly higher in the D+/R− group than in the D–/R–and R+ groups (16.6% vs. 11.8% vs. 14.4%, respectively; *P* = 0.02). The D+/R− group was also hospitalized significantly more frequently than the D–/R–and R+ groups (67.2% vs. 60.0% vs. 63.0%, respectively; *P* = 0.005).

**TABLE 2:** One-year outcomes post transplant^a^.

Outcome	D–/R–	R+	D+/R–	*P* value
Kidney rejection	45 (6.4) (n = 700)	163 (7.3) (n = 2,244)	96 (9.2) (n = 1,040)	0.06
Pancreas rejection	51 (7.6) (n = 668)	192 (9.0) (n = 2,134)	103 (10.4) (n = 986)	0.14
Kidney or pancreas rejection	79 (11.8) (n = 669)	308 (14.4) (n = 2,144)	163 (16.6) (n = 980)	0.02
Hospitalization	437 (60.0) (n = 728)	1,470 (63.0) (n = 2,334)	737 (67.2) (n = 1,097)	0.005

Abbreviations: D–/R–, CMV-negative donors and recipients; D+/R–, CMV-positive donors and CMV-negative recipients; R+, CMV-positive recipients.

^a^ Values are No. of patients (%) or mean (SD).

**FIGURE 2:**
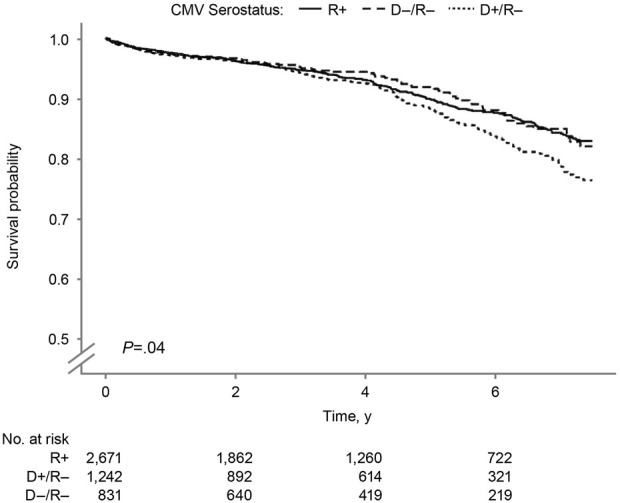
Recipient Survival by Donor-Recipient CMV Serostatus. Kaplan–Meier curves showing recipient survival after SPK transplantation according to donor–recipient CMV serostatus. CMV indicates cytomegalovirus; R+, CMV-positive transplant recipients; D+/R–, CMV-positive donors and CMV-negative recipients; D–/R–, CMV-negative donors and recipients.

In the Kaplan-Meier analysis of recipient survival (Figure 2), the D+/R− group had the lowest overall survival (log-rank *P* = 0.03). The 7.5-year survival probabilities were 82.1%, 82.9%, and 76.4% in the D−/R−, R+, and D+/R–groups.

**FIGURE 3:**
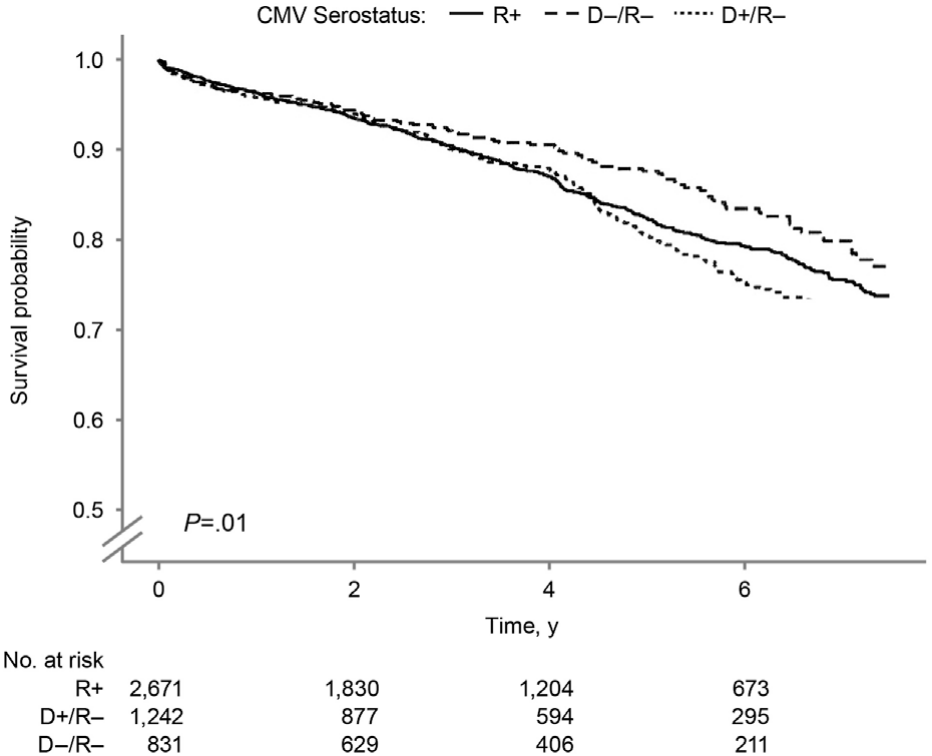
Overall Kidney Graft Survival by Donor-Recipient CMV Serostatus. Kaplan–Meier curves for overall kidney graft survival by donor–recipient CMV serostatus among SPK transplant recipients. CMV indicates cytomegalovirus; R+, CMV-positive transplant recipients; D+/R–, CMV-positive donors and CMV-negative recipients; D–/R–, CMV-negative donors and recipients.

**FIGURE 4:**
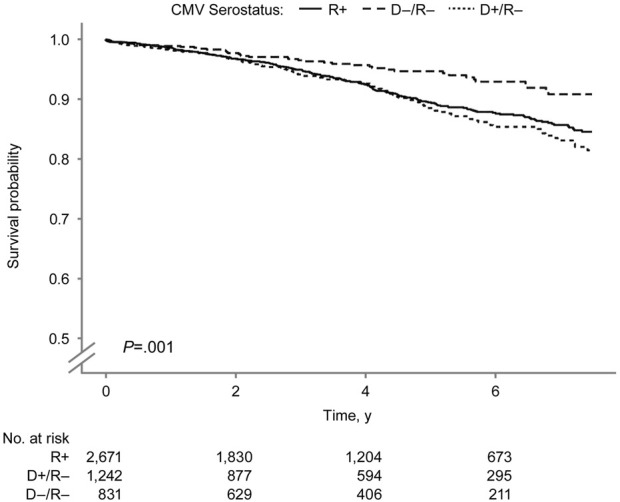
Death-censored Kidney Graft Survival by Donor-Recipient CMV Serostatus. Death-censored kidney graft survival after SPK transplantation by donor–recipient CMV serostatus. CMV indicates cytomegalovirus; R+, CMV-positive transplant recipients; D+/R–, CMV-positive donors and CMV-negative recipients; D–/R–, CMV-negative donors and recipients.

The overall kidney survival probability (Figure 3) was significantly lower in the D+/R− group than in other groups (log-rank *P* = 0.01). The 7.5-year overall kidney survival probabilities were 77.1%, 73.8%, and 68.1% in the D−/R−, R+, and D+/R–groups. Figure 4 shows the death-censored survival of kidney allografts. The D−/R− group had a significantly higher probability of graft survival than the other groups (log-rank *P =* 0.001). The 7.5-year death-censored kidney graft survival probabilities were 90.8%, 84.6%, and 81.5% in the D−/R−, R+, and D+/R–groups.

**FIGURE 5:**
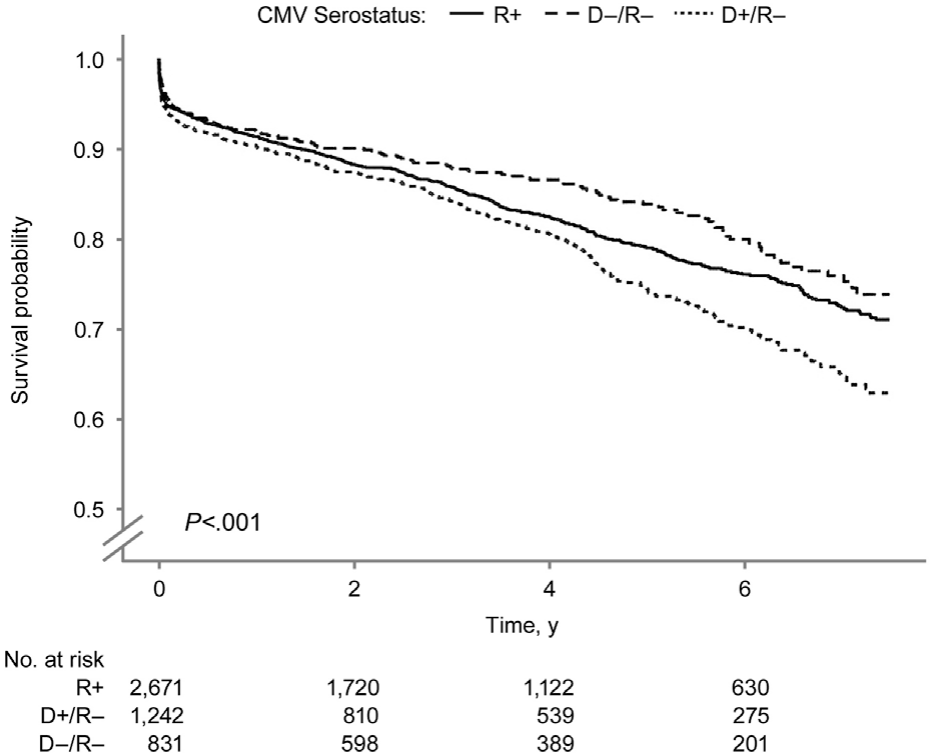
Overall Pancreas Graft Survival by Donor-Recipient CMV Serostatus. Overall pancreas graft survival after SPK transplantation by donor–recipient CMV serostatus. CMV indicates cytomegalovirus; R+, CMV-positive transplant recipients; D+/R–, CMV-positive donors and CMV-negative recipients; D–/R–, CMV-negative donors and recipients.

**FIGURE 6:**
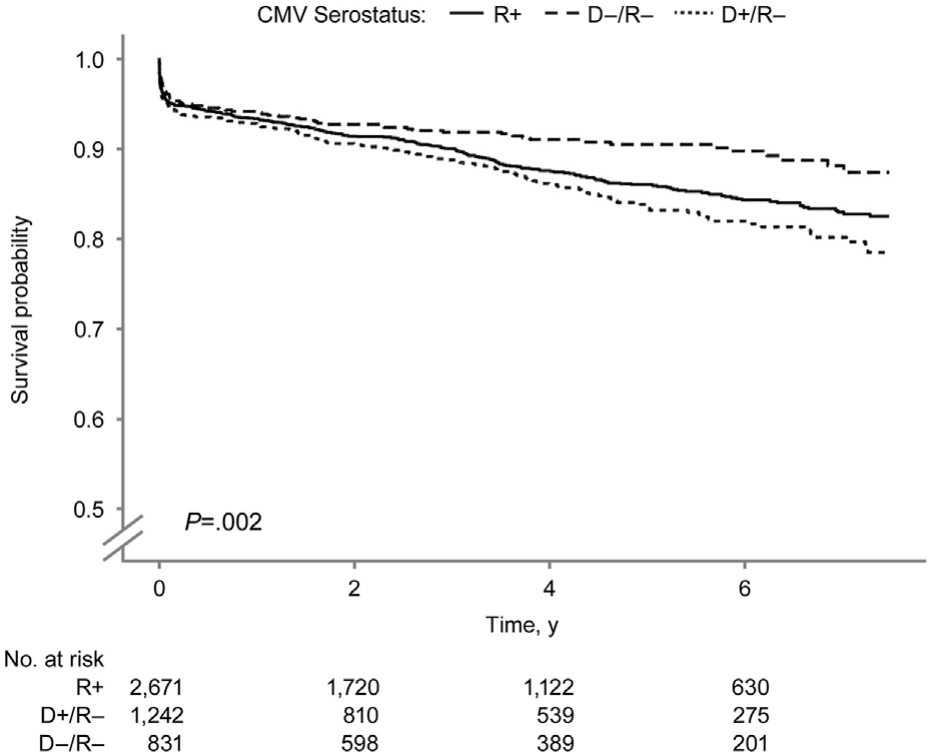
Death-censored Pancreas Graft Survival by Donor-Recipient CMV Serostatus. Death-censored pancreas graft survival by donor–recipient CMV serostatus among SPK transplant recipients. CMV indicates cytomegalovirus; R+, CMV-positive transplant recipients; D+/R–, CMV-positive donors and CMV-negative recipients; D–/R–, CMV-negative donors and recipients.

The overall pancreas allograft survival probability (Figure 5) was significantly lower in the D+/R− group (log-rank *P* < 0.001). The 7.5-year overall pancreas survival probabilities were 73.8%, 71.0%, and 62.8% in the D−/R−, R+, and D+/R–groups. Figure 6 shows the death-censored pancreas graft survival probabilities. The D−/R− group had a significantly higher probability of graft survival than the other groups (log-rank *P* = 0.002). The 7.5-year death-censored pancreas allograft survival probabilities were 87.3%, 82.5%, and 78.4% in the D−/R−, R+, and D+/R–groups.

### Multivariable Outcomes

In the multivariable model for recipient death and graft loss, compared with R+ serostatus, D+/R− serostatus was significantly associated with a 28% higher risk of death (*P* = 0.045) and a 25% higher risk of pancreas graft loss (*P* = 0.009) (Table 3). It was also associated with a 20% higher risk of kidney graft loss, but the effect was not significant (*P* = 0.06). CMV D−/R− serostatus was not associated with altered risks of death or pancreas or kidney graft loss. However, in the death-censored graft loss model, D−/R− serostatus was significantly associated with a 25% lower risk of pancreas graft loss (*P* = 0.04), and a 34% lower risk of kidney graft loss (*P* = 0.03), compared with R+ serostatus. In the death-censored model, D+/R–serostatus was significantly associated with a 31% higher risk of kidney graft loss (*P* = 0.04) compared with R+ serostatus.

**TABLE 3:** Multivariable cox proportional hazard models for recipient, pancreas, and kidney outcomes^a^ ^,^ ^b^.

Outcome	D+/R–		D–/R–	
	Hazard ratio (95% CI)	*P* value	Hazard ratio (95% CI)	*P* value
Overall outcomes				
Death	1.28 (1.01–1.62)	0.045	0.98 (0.73–1.32)	0.89
Pancreas graft loss	1.25 (1.06–1.48)	0.009	0.86 (0.70–1.07)	0.17
Kidney graft loss	1.20 (1.00–1.46)	0.06	0.85 (0.66–1.09)	0.19
Death-censored outcomes				
Pancreas graft loss	1.19 (0.96–1.47)	0.11	0.75 (0.56–0.99)	0.04
Kidney graft loss	1.31 (1.01–1.69)	0.04	0.66 (0.46–0.96)	0.03

Abbreviations: D–/R–, CMV-negative donors and recipients; D+/R–, CMV-positive donors and CMV-negative recipients.

^a^ Models were adjusted for recipient age, sex, ethnicity, diabetes type, preemptive transplant, years on dialysis, induction type, corticosteroid maintenance, HLA, antigen mismatch, calculated panel reactive antibody, local vs. imported organs, pancreas donor risk index, and donor-recipient Epstein-Barr virus status.

^b^ D+/R− and D−/R− groups were each compared with the R+ group (CMV-positive recipients) using the same model.

### Causes of Death and Graft Failure

The causes of death and kidney and pancreas allograft failure are detailed in Supplementary Tables S1–S3. Notably, the specific cause of death was not reported for most recipients. Cancer as a cause of death was reported more frequently in the D+/R–group than in the other groups (Supplementary Table S1).

The causes of kidney and pancreas graft failure were not reported for approximately half of the recipients. Kidney graft rejection was the leading documented cause of kidney graft loss in the D+/R− group (Supplementary Table S2). Primary nonfunction was the most common documented cause of pancreas graft loss (Supplementary Table S3). Pancreas rejection was the cause of graft loss more frequently in the D+/R− group than in the other groups.

## Discussion

Our analysis of the SRTR database represents the most contemporary report on the outcomes of SPK transplant recipients stratified by donor-recipient CMV serostatus. Our results highlight significant differences in clinically meaningful short- and long-term outcomes for CMV-naïve patients, depending on whether they received allografts from a CMV-seropositive or CMV-seronegative donor. CMV D+/R− serostatus was associated with higher risks of death and overall graft loss, and CMV D−/R− status was associated with lower risks of death-censored kidney and pancreas graft loss. The rates of hospitalization and combined kidney or pancreas rejection were significantly higher in the CMV D+/R− group and lower in the CMV D−/R− group.

CMV is known to confer worse outcomes after solid-organ transplant, both directly by invading the organ allograft and indirectly by increasing the risk of rejection, promoting immune suppression, and predisposing the recipient to other infections and complications. Conversely, the absence of CMV infection in transplant recipients (D–/R–) may confer several clinical benefits. First, it eliminates the many indirect viral effects (including CMV-associated rejection, secondary opportunistic infections, and virus-induced inflammation) that can contribute to long-term allograft dysfunction [17, 32]. Second, it obviates the need to reduce immunosuppression, thereby minimizing the risk of rejection, which is often exacerbated when immunosuppression must be tapered to control CMV infection [24]. Third, recipients with CMV D–/R–serostatus do not need prolonged antiviral prophylaxis or therapy and thus avoid the adverse hematologic effects associated with valganciclovir (such as leukopenia, neutropenia, and bone marrow suppression), which can predispose them to secondary infections and graft complications [33]. Our findings showed that CMV D−/R− serostatus was associated with better graft survival, reduced morbidity, and better long-term patient outcomes in SPK transplant recipients.

To reduce the risk of adverse outcomes associated with CMV, prophylaxis with valganciclovir is recommended as the standard of care for high-risk CMV D+/R− solid-organ transplant recipients, as well as those with augmented immunosuppression after organ transplant (such as all at-risk lung and pancreas transplant recipients) [17]. During the study period, valganciclovir prophylaxis was routinely administered to R+ recipients for 3 months and to D+/R–recipients for up to 6 months [17, 23]. However, valganciclovir prophylaxis is often associated with leukopenia, which may require 1) an adjustment in immunosuppression (e.g., reduction in dose of mycophenolate mofetil) which can then increase the risk of rejection or 2) discontinuation of either trimethoprim-sulfamethoxazole prophylaxis or valganciclovir prophylaxis which can then increase the risk of infections. The magnitude of these adverse events is not negligible. In one study, 53% of D+/R–SPK recipients had leukopenia during valganciclovir prophylaxis, resulting in reduced immunosuppression in most recipients and discontinuation of the valganciclovir prophylaxis in more than 28% of recipients [19].

Individualization of immunosuppressive protocols has been explored as a strategy to reduce infectious complications, including CMV infection, particularly through selective use of non-depleting induction agents [34, 35] or mTOR inhibitors [36, 37]. Our group had previously examined the outcomes of SPK recipient by induction type [38] and non-depletions induction had similar results to r-ATG in terms of recipient and grafts survival. Although tailored approaches may decrease the incidence of CMV and other posttransplant infections, existing evidence suggests that these modifications have not translated into improved recipient or graft survival after SPK transplantation. In our cohort, outcomes did not differ significantly by induction type, and CMV donor–recipient serostatus discordance (D+/R−) remained the primary determinant of adverse outcomes. This persistent disparity despite efforts to personalize immunosuppression underscores the substantial, independent impact of CMV serostatus on long-term outcomes and highlights the need for national and global initiatives aimed at mitigating the risks associated with high-risk CMV mismatches.

Another challenge for SPK recipients is the risk of delayed-onset CMV infection after discontinuing valganciclovir prophylaxis [22]. Ahopelto et al [19] reported a 68% rate of primary CMV infection among CMV D+/R− SPK transplant recipients in Finland, mainly after the conclusion of 6 months of valganciclovir prophylaxis. In contrast, 36% of CMV R+ recipients had CMV infection after completing 3 months of valganciclovir prophylaxis. The rates of hospitalization and recurrent, refractory, and resistant cases were 2- to 4-fold higher in CMV D+/R− patients than in CMV R+ patients. Our results complement these findings and underscore the current challenges involved in treating SPK transplant recipients. Even in an era of prolonged prophylaxis (up to 6 months for high-risk patients), the negative effects of CMV on short- and long-term allograft and patient survival remain a substantial challenge.

The association between CMV serostatus and posttransplant allograft and patient outcomes has been shown for other organ transplant types. In a study of kidney-alone transplants, Leeaphorn et al [39] reported that D+/R− serostatus was associated with a 17% higher risk of kidney graft loss and an 18% higher risk of death. Lockridge and colleagues [40] adopted an innovative policy change in an Oregon organ procurement organization that allowed for matching on the basis of donor-recipient CMV status, with some exceptions. This policy change aimed to reduce the number of high-risk D+/R− transplants and increase the number of low-risk D−/R− transplants. The resulting variance in allocation was not associated with changes in transplant rates in either group. However, the national kidney and pancreas allocation systems do not consider CMV matching. Axelrod et al [41] found that D–/R–serostatus was associated with better kidney graft survival, more quality-adjusted life years, and lower costs than D+/R–serostatus. Moreover, they modeled the outcomes of recipients who had to wait for a CMV-negative donor and found survival benefits of up to 30 months. Our results support the findings of these other groups and expand the potential benefits of CMV matching to SPK recipients. However, further studies are needed to assess the practicality and the broader impact of allocation changes.

### Strengths and Limitations

To our knowledge, this study represents the largest and most comprehensive to date in documenting the long-term outcomes of SPK transplant recipients on the basis of donor-recipient CMV risk profiles. It was designed to isolate the effects of donor-recipient CMV risk profiles by including only conventional-risk recipients with crossmatch-negative transplants and by using a standardized maintenance regimen. Primary outcomes were based on well-documented metrics from the SRTR.

However, the study has limitations. First, the retrospective design prevents full adjustment for unmeasured confounders. Second, the SRTR standard analysis file has substantial variability in center reporting practices. For example, although the cohort was limited to recipients discharged on a standard maintenance regimen of tacrolimus and mycophenolate mofetil, the standard analysis file lacks consistent data on postdischarge changes to immunosuppressive regimens. This restricts the ability of researchers to analyze variances in immunosuppression exposure, or to assess tolerability of SPK transplant recipients to immunosuppression. Third, the SRTR does not capture granular longitudinal data on the duration of CMV prophylaxis, the magnitude of CMV infection (e.g., viral load) or disease, antiviral drug resistance or management, donor-specific antibody formation, or late rejection episodes. Therefore, it is difficult to determine whether the outcomes observed in this study were primarily influenced by active CMV infection, given that delayed-onset CMV infection remains a common phenomenon in SPK transplant [19, 22]. Finally, the lack of access to a biorepository or T-cell profiling limits the ability of researchers to examine the relationship between primary CMV infection and the immune system in SPK transplant recipients.

### Conclusion

In this large cohort of SPK transplant recipients, having a high-risk donor-recipient CMV serostatus discordance (D+/R−) was associated with a significantly higher risk of death and overall graft loss. In contrast, concordant-negative CMV serostatus (D−/R−) was associated with significantly higher death-censored survival of both kidney and pancreas grafts. The high-risk group also had the highest rates of complications, including rejection and hospitalization, whereas the low-risk group had significantly lower rates of these outcomes.

These findings underscore the potential benefit of matching CMV-seronegative transplant recipients with organs from CMV-seronegative donors. Implementing such matching strategies could improve overall survival rates for recipients and allografts and help with CMV prevention. However, further research is needed to evaluate the potential effects of extending wait times for seronegative organs and to explore the feasibility of revising the allocation policies.

## Data Availability

The data analyzed in this study is subject to the following licenses/restrictions: All relevant data supporting the findings of this study are reported within the article or available from the SRTR database, subject to the data use agreement. Requests to access these datasets should be directed to SR, riad.samy@mayo.edu.

## Abbreviations

CMV, cytomegalovirus; D+/R–, donor positive and recipient negative; D–/R–, donor negative and recipient negative; R+, recipient positive; SPK, simultaneous pancreas-kidney; SRTR, Scientific Registry of Transplant Recipients.
