# Supplementary material for: Death and Graft Loss in Simultaneous Pancreas-Kidney Recipients by Donor-Recipient Cytomegalovirus Serostatus in the United States
Source: Transpl Int. 2025 Nov 26;38:15653. doi: 10.3389/ti.2025.15653 (PMC12690930; doi:10.3389/ti.2025.15653)
Supplement: Supplementary file 1 [file Table1.docx]

**Supplementary Table 1.** Causes of death by donor-recipient CMV serostatus

|  | **R+** | **D+/R-** | **D-/R-** |
| --- | --- | --- | --- |
|  | (n=232) | (n=140) | (n=72) |
|  |  |  |  |
| Vascular disease | 33 (17.9%) | 19 (17.2%) | 11 (19.3%) |
| Trauma or hemorrhage | 10 (5.5%) | 4 (3.6%) | 1 (1.8%) |
| Cancer | 14 (7.6%) | 10 (9.1%) | 3 (5.3%) |
| Infection | 16 (8.7%) | 12 (10.9%) | 9 (15.8%) |
| Unknown/Miscellaneous | 86 (46.7%) | 50 (45.5%) | 30 (52.6%) |
| Other | 25 (13.6%) | 15 (13.6%) | 3 (5.3%) |
| Not reported | 48 | 30 | 15 |

Percentages do not reflect causes not reported.

**Supplementary Table 2.** Causes of kidney graft failure by donor-recipient CMV serostatus

|  | **R+** | **D+/R-** | **D-/R-** |
| --- | --- | --- | --- |
|  | (n=210) | (n=113) | (n=41) |
|  |  |  |  |
| Acute or chronic rejection | 49 (59.0%) | 32 (65.3%) | 7 (46.7%) |
| Infection or BK virus | 4 (4.8%) | 2 (4.0%) | 0 (0.0%) |
| Primary non-function | 10 (12.0%) | 2 (4.1%) | 1 (6.7%) |
| Recurrent disease | 2 (2.4%) | 0 (0.0%) | 2 (13.3%) |
| Other | 18 (21.7%) | 13 (26.5%) | 5 (33.3%) |
| Not reported | 127 | 64 | 26 |

Percentages do not reflect causes not reported. Other causes include pancreatitis and bleeding.

**Supplementary Table 3.** Causes of pancreas graft failure by donor-recipient CMV serostatus

|  | **R+** | **D+/R-** | **D-/R-** |
| --- | --- | --- | --- |
|  | (n=316) | (n=172) | (n=75) |
|  |  |  |  |
| Acute or chronic rejection | 35 (19.9%) | 23 (22.5%) | 6(14%) |
| Anastomotic leak | 7 (4.0%) | 4 (3.9%) | 2 (4.7%) |
| Primary non-function or thrombosis | 87 (49.4%) | 52 (51.0%) | 23 (53.5%) |
| Infection | 6 (3.4%) | 1 (1.0%) | 0 (0.0%) |
| Other* Pancreatitis, bleeding | 41 (23.3%) | 22 (21.6%) | 12 (27.9%) |
| Not reported | 140 | 70 | 32 |

Percentages do not reflect causes not reported.
